# Association of microvascular biomarkers in fluorescein angiography with macrovascular-related mortality in clinical routine data

**DOI:** 10.1371/journal.pone.0266423

**Published:** 2022-05-05

**Authors:** Felix Goldbach, Georgios Mylonas, Martin Riegelnegg, Jonas Brugger, Ursula Schmidt-Erfurth, Bianca S. Gerendas

**Affiliations:** 1 Department of Ophthalmology and Optometry, Medical University of Vienna, Vienna, Austria; 2 Center for Medical Statistics, Informatics, and Intelligent Systems (CeMSIIS), Medical University of Vienna, Vienna, Austria; Nankai University, CHINA

## Abstract

**Purpose:**

Early detection of microvascular changes in the retina may be important for the risk assessment of cardiovascular health. Therefore, the purpose of this study was to investigate imaging biomarkers in fluorescein angiography (FA) as potential predictors for cardiovascular mortality.

**Methods:**

In this retrospective, matched case-control study, we included FA images from clinical routine data between 2007 and 2018 of 100 patients who died of macrovascular events (Group 1) and 100 age- and sex-matched controls (Group 2). All patients were under treatment for different, mostly retinal, ocular diseases. FA images were used for the measurement of the foveal avascular zone (FAZ) and the arteriolar and venular caliber.

**Results:**

Patients mean age on examination day was 69.5 ± 8.3 years with a 1:1 female:male subject ratio. Mean FAZ area of our sample was 0.340 ± 0.135 mm^2^ for Group 1 and 0.264 ± 0.137 mm^2^ for Group 2 (P < 0.001), showing a larger FAZ area in patients who subsequently died of macrovascular-related systemic diseases.

**Conclusions:**

Individuals effected by a macrovascular-related disease show a larger FAZ on FA examinations before the event compared to patients which are unaffected. Our results highlight a possible role of the FAZ as additional biomarker for the cardiovascular condition.

## Introduction

Cardiovascular diseases (CVDs) such as ischemic heart disease and stroke have been the leading cause of mortality in the world for 15 years; claiming 17.79 million lives a year [1]. It is expected that the numbers will steadily grow, due to the ageing population and modifiable risk factors such as unhealthy diet and physical inactivity [2]. Major traditional risk factors of CVD include ApoB/ApoA1 ratio—5 vs 1 (OR 3.25), smoking (OR 2.87), diabetes (OR 2.37), hypertension (1.91), and abdominal obesity (OR 1.62) [3]. The liability of cardiovascular diseases leads to human suffering, disability, reduced productivity, increasing health care costs and numerous other consequences that have resulted in a strengthened focus on risk assessment and primary prevention strategies of CVDs [4–6]. For composition and implementation of preventive measures it is essential to identify individuals at risk of CVDs [7]. But traditional risk factors are still insufficient to predict CVD events precisely.

However, the retinal microvasculature offers an easily accessible insight into the condition of the human microcirculation. McClintic et al. hypothesized that as retinal vessels have approximately the same magnitude as the coronary microvasculature (~100–250μm), they could serve as a marker for subclinical or microvascular coronary disease [8]. Therefore, early detection of alterations in retinal microvasculature might be an opportunity for the risk assessment of cardiovascular mortality. These alterations can be observed using various methods, including fundus examination (FE), optical coherence tomography angiography (OCTA) and fluorescence angiography (FA).

Previous studies have examined the relationship between retinal microvascular alterations and CVD using different retinal imaging biomarkers such as the arteriolar and venular caliber, arteriovenous nicking, arteriolar tortuosity, or vessel density. Another significant retinal imaging biomarker is the foveal avascular zone (FAZ). The FAZ is the central retinal area and as such the most relevant retinal area for central visual function. It is surrounded by interconnected capillary networks, which allow its easy identification. This region is highly sensitive to ischemic events and analyzing its morphology can provide insights into potential pathologic processes [9]. An enlargement of the FAZ area due to ischemia, has been found in diabetic retinopathy [10], retinal vein occlusion [11] and arterial hypertension [12, 13]. These studies support the claim of an association between retinal microvasculature alterations and CVD as the mentioned diseases are risk factors for CVD as well. Despite the obvious success of non-invasive OCTA examinations nowadays, FA is still considered the gold standard for imaging the retinal (micro)-vasculature in many cases. Furthermore, FA is widely available, it has a good sensitivity when detecting slow blood flow vessels [14] and arterioles and venules are clearly visible and can therefore be distinguished from each other very well. This study was performed to assess changes in the retinal microvasculature of patients who died from a cardiovascular event by comparing FA imaging biomarkers with age- and sex matched patients who are still alive. This may contribute to the prediction and prevention of CVD associated mortality.

## Methods

This retrospective matched case-control study was approved by the ethics committee of the Medical University of Vienna (Application number 2094/2018 and 2095/2018) and was conducted in accordance with the declaration of Helsinki. As approved by the Ethics committee of the Medical University of Vienna no informed consent was necessary for this retrospective analysis.

### Study population

Using the Austrian death registry, all patients, whose death was caused by a cardiovascular-related disease by 12/2018 and who had had an FA examination at our department, could be determined. Patients with the following causes of death were included in the study: myocardial infarction, ischemic cardiomyopathy, apoplexy, pulmonary embolism and embolization or thrombosis of another region. Patients were excluded if any retinal disease obscured the entire foveal area and/or the boundaries of the FAZ or if poor image quality no longer allowed identification of vessels around the optic disc or the FAZ itself. All patients, who underwent fluorescein angiography examinations at the Department of Ophthalmology and Optometry of the Medical University of Vienna between 01/2007 and 12/2018 were pooled. From this pool of 6493 available patients with FA examinations, 460 patients whose death was caused by a cardiovascular-related disease were identified. 50 males and 50 females (Group 1) of these 460 patients were randomly chosen by an ophthalmologist using a random number generator. If exclusion criteria were not met, the patients were included in the study. During this process 140 patients were excluded due to either poor image quality or ungradable FA examinations. From the remaining pool of 6033 patients, all included patients of Group 1 were randomly age- and sex-matched with alive patients. These patients must still have been alive in 12/2018 (Group 2) and must have had available FA examinations at the same age (full year at examination date) as patients of Group 1. During this process 98 patients were excluded due to either poor image quality or ungradable FA examinations. Collectively, 200 patients were enrolled in this study. Included patients were under treatment for different, mostly retinal, ocular diseases. Prescence of diabetes and all ocular diseases of the study eye assigned at the time of the FA examination were recorded.

### Image acquisition and analysis

Images were chosen from the FA image pool of the mentioned period. All FA examinations had been performed with a Heidelberg SPECTRALIS HRA+OCT camera (Heidelberg Engineering Inc., Heidelberg, Germany) by experienced ophthalmologists or optometrists according to a standardized protocol during regular outpatient clinic consultations. For each patient, an early phase fovea centered FA image (Field 2) and an optic disc centered image (Field 1) after 5 minutes with the best available image quality were selected and exported as TIF file. All exported images were evaluated for retinal vascular alterations using the image processing program ImageJ 1.52a. In addition, a retinal specialist graded all FA examinations for presence of diabetic retinopathy to identify this common disease as a potential confounder.

### Foveal avascular zone area

**Fig 1:**
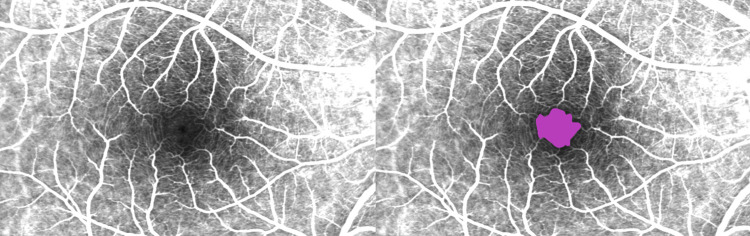
Optimized fluorescein angiography image. Brightness and image contrast optimization in an early phase fluorescein angiography image (Field 2) without and with annotated FAZ area (magenta).

The measurement of the FAZ using the early phase Field 2 images were performed by two independent, experienced, and certified image graders of the Vienna Reading Center according to a predefined standardized grading procedure. Prior to the analysis, brightness and image contrast were optimized to ensure the best possible representation of the FAZ (Fig 1). Using the image scale, the pixel per μm ratio could be determined and set as a global scale. All FAZ areas were marked by the two graders and their values were noted (in μm^2^) for later evaluation.

### Generalized arteriolar and venular caliber

**Fig 2:**
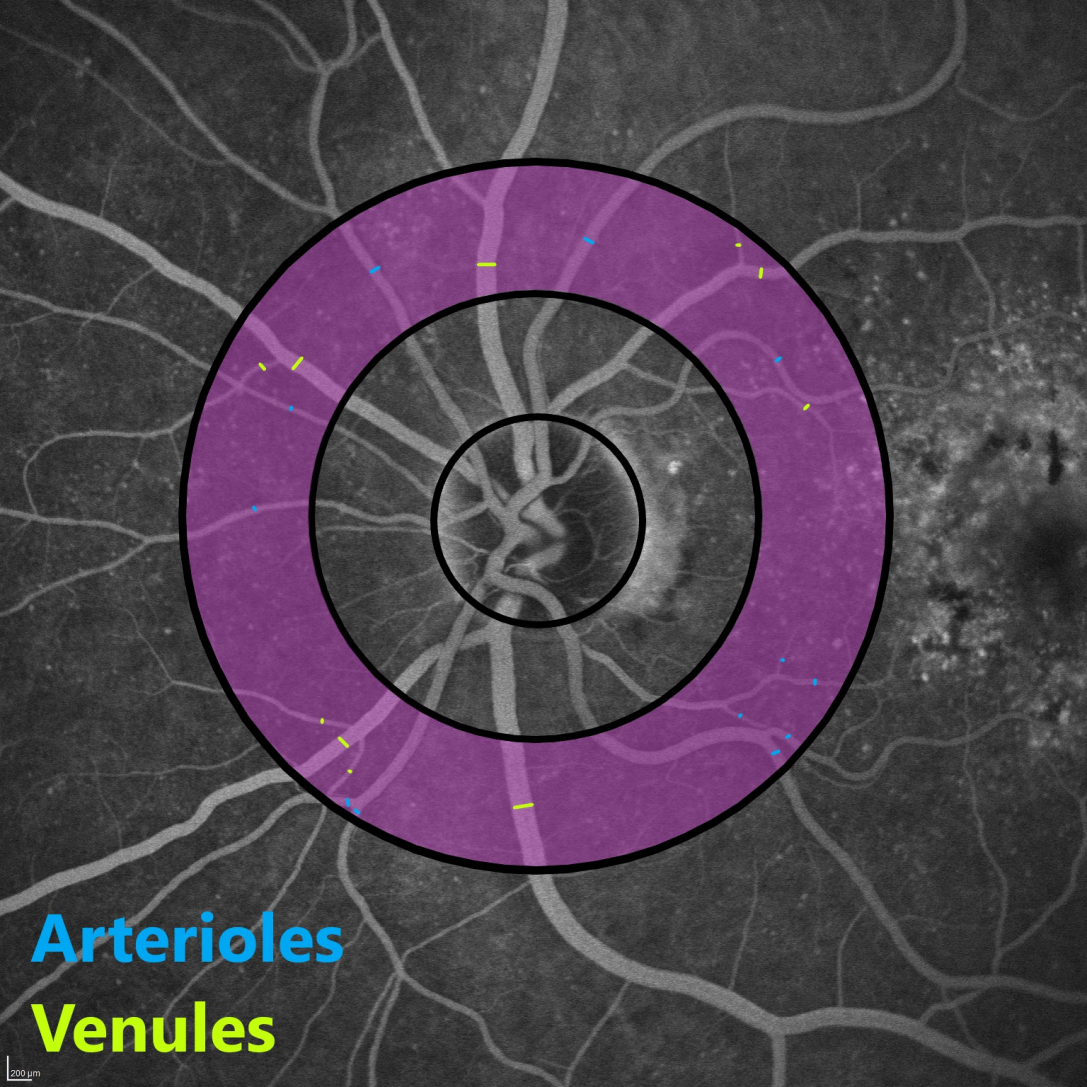
Measurement zone. Optic disc centered fluorescein angiography image (Field 1) at minute five with overlaid grid. The grid is composed of three circles concentric with the optic disc: The innermost circumscribes the average disc, the middle one bounds the annulus from the disc margin to 0.5 disc diameter (DD) from the margin, and the outer one bounds the annulus from 0.5 DD to 1 DD from the disc margin. The measurement zone (magenta) is delimited by the middle and outer circle. Measurements of the arterioles are displayed in blue, venules in green.


The method used for the assessment of the vessels around the optic disc was adapted from Hubbard et al. [15] for FA. All measurements and calculations were performed by one experienced image grader. For each Field 1 image, the diameter of the optic disc was determined. As a next step the FA image was overlaid by the grader with a scalable grid to establish the measurement zone (Fig 2). The grader identified arterioles and venules before measuring the diameter of each vessel coursing within the zone. Thereafter, the grader chose the segment of each vessel within the measurement zone most suitable for measurement based on image quality and straightness of the vessel. All measurements were taken at a rectangular angle to the vessel course to determine the true caliber of each vessel. Measurements of individual arterioles and venules were later combined into a central retinal arteriolar equivalent (CRAE) and a central retinal venular equivalent (CRVE) representing the mean arteriolar or venular caliber of the patients’ eye. Calculations were performed according to the formulas developed by Parr et al. [16, 17] and Hubbard et al. [18] as seen in Eq (1). The formulas summarize all arterioles and venules into one representative value. For details, please refer to the description of Eq (1).

$$ArteriolesC_{R}=0.87C_{s}{}^{2}+1.01C_{l}{}^{2}–0.22C_{s}C_{l}-10.76$$

$$VenulesC_{R}=0.72C_{s}{}^{2}+0.91C_{l}{}^{2}+450.05$$

Eq (1) Formula for central retinal arteriolar/venular equivalent (CRAE/CRVE).

CR is the caliber of throot vessel, Cs the caliber of the smaller branch and Cl the caliber of the larger branch. Using the formula, the largest arteriole was combined with the smallest arteriole into a new root arteriole (CR), then the second largest and second smallest were combined and so on until all were accounted for. If the number of arterioles to be combined was odd, the remaining arteriole was carried over to the next iteration. The pairing process was repeated until one last root arteriole was obtained, representing the CRAE. The procedure for the root venule (CRVE) was performed accordingly. To quantify generalized narrowing or widening, CRAE and CRVE were obtained for all eyes and their Arteriolar-Venular-Ratio (AVR) was calculated. An AVR of 1 signifies that arteriolar and venular calibers are on average the same. A lower AVR suggests either relatively narrower arterioles than venules or relatively wider venules than arterioles.

### Statistical analysis

To determine whether the patient groups differed in terms of size of their FAZ, CRAE, CRVE or Arteriolar-Venular-Ratio (AVR) at their examination date a paired t-test was performed, where pairs were matched with regards to gender and age. Following this, a subgroup analysis dependent on gender for FAZ, CRAE, CRVE and AVR was performed using paired t-tests. Continuous data within the subgroup were normally distributed. A standard logistic regression model and a conditional logistic regression model with the status of a person as the dependent and FAZ area as the independent variable were computed. Logistic regression models were adjusted for ocular diseases, presence of diabetes and presence of diabetic retinopathy. The strata in the conditional logistic model were defined by the matched pairs. ROC curves were computed to assess the diagnostic ability of the logistic models. Pearson-Chi-Quadrat test was applied to determine whether the groups differed in terms of presence of diabetes, graded diabetic retinopathy or their assigned ocular diagnoses. Bland-Altmann-Diagrams were created to assess the agreement of the image graders that evaluated the size of the FAZ. All parameters with a p-value smaller than 0.05 were considered as statistically significant. All calculations were performed using the statistical software R version 3.5.3 or higher.

## Results

### Subjects

**Fig 3:**
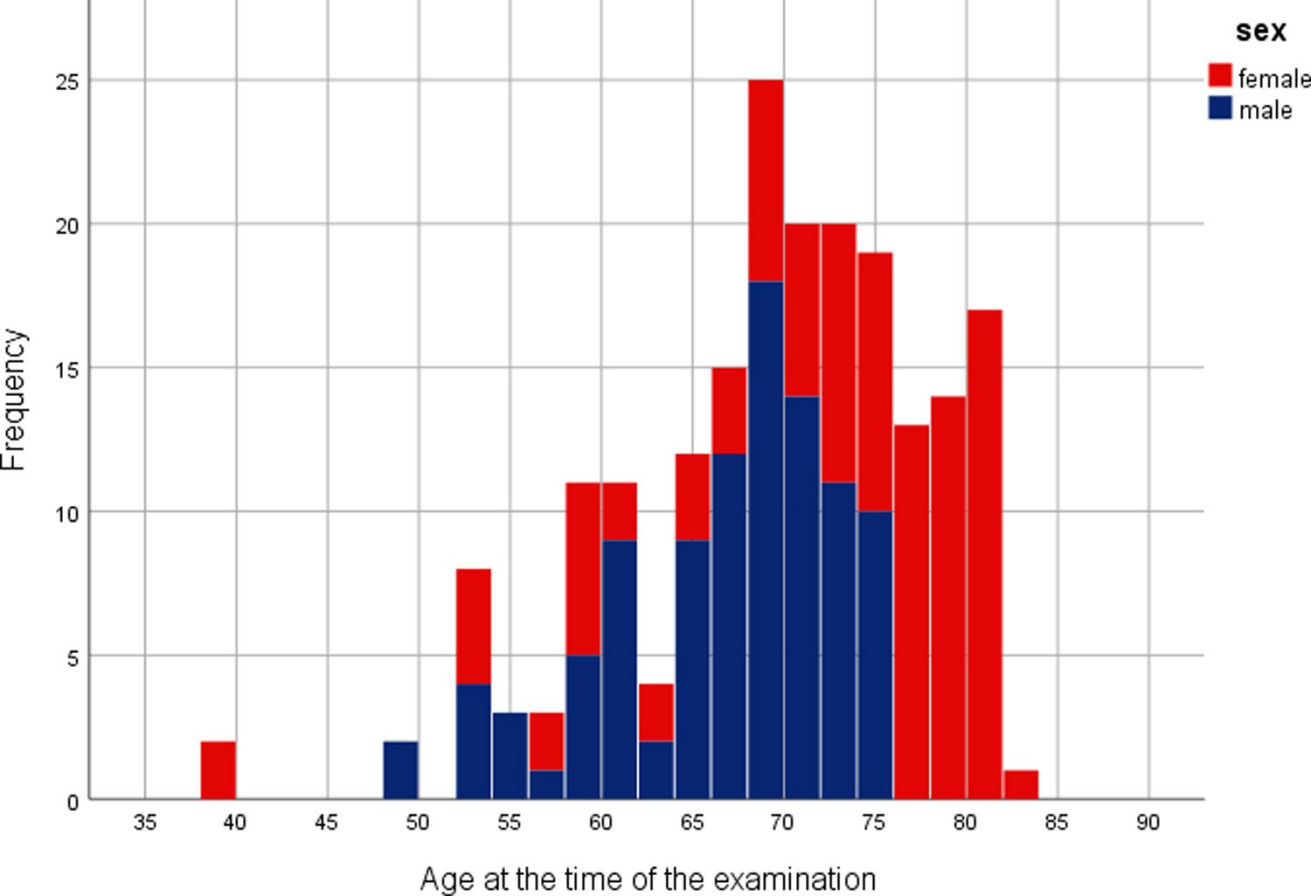
Age distribution. Age distribution of the study population at the time of the examination considering gender. Female patients are displayed in red, male patients in blue.

200 FA examinations were analyzed by the Vienna Reading Center. The mean age on the examination day was 69.5 ± 8.3 years (Fig 3). A total of 109 right eyes and 91 left eyes were evaluated. There were no significant differences between Group 1 and Group 2 regarding assigned ocular diagnoses, presence of diabetic retinopathy or presence of diabetes (Table 1).

**Table 1:** Distribution of graded diabetic retinopathy and assigned (ocular) diagnoses*.

Grading / Diagnosis	Group 1	Group 2	p Value
Diabetic Retinopathy (%)**	35	23	0.980
Diabetes (%)	56	23	0.592
Neovascular age-related macular degeneration (%)	13	21	0.097
Dry age-related macular degeneration (%)	12	25	0.155
Retinal vein occlusion (%)	2	5	0.743
Glaucoma (%)	5	4	0.640
Uveitis / Vasculitis (%)	5	4	0.640
Other diagnose*** (%)	21	5	0.981
No diagnose (%)	4	7	0.575

*Patients may have had more than one assigned (ocular) diagnosis for the study eye.

**As graded by Retinal Specialist on fluorescein angiography examination.

***Other diagnoses include rarely assigned diagnoses like Hypertensive retinopathy, Macular pucker, Posterior vitreous detachment, Bleedings, Irvine–Gass syndrome, Radiation retinopathy, Pattern dystrophy, MacTel 2 and Central serous chorioretinopathy.

### Analysis

**Fig 4:**
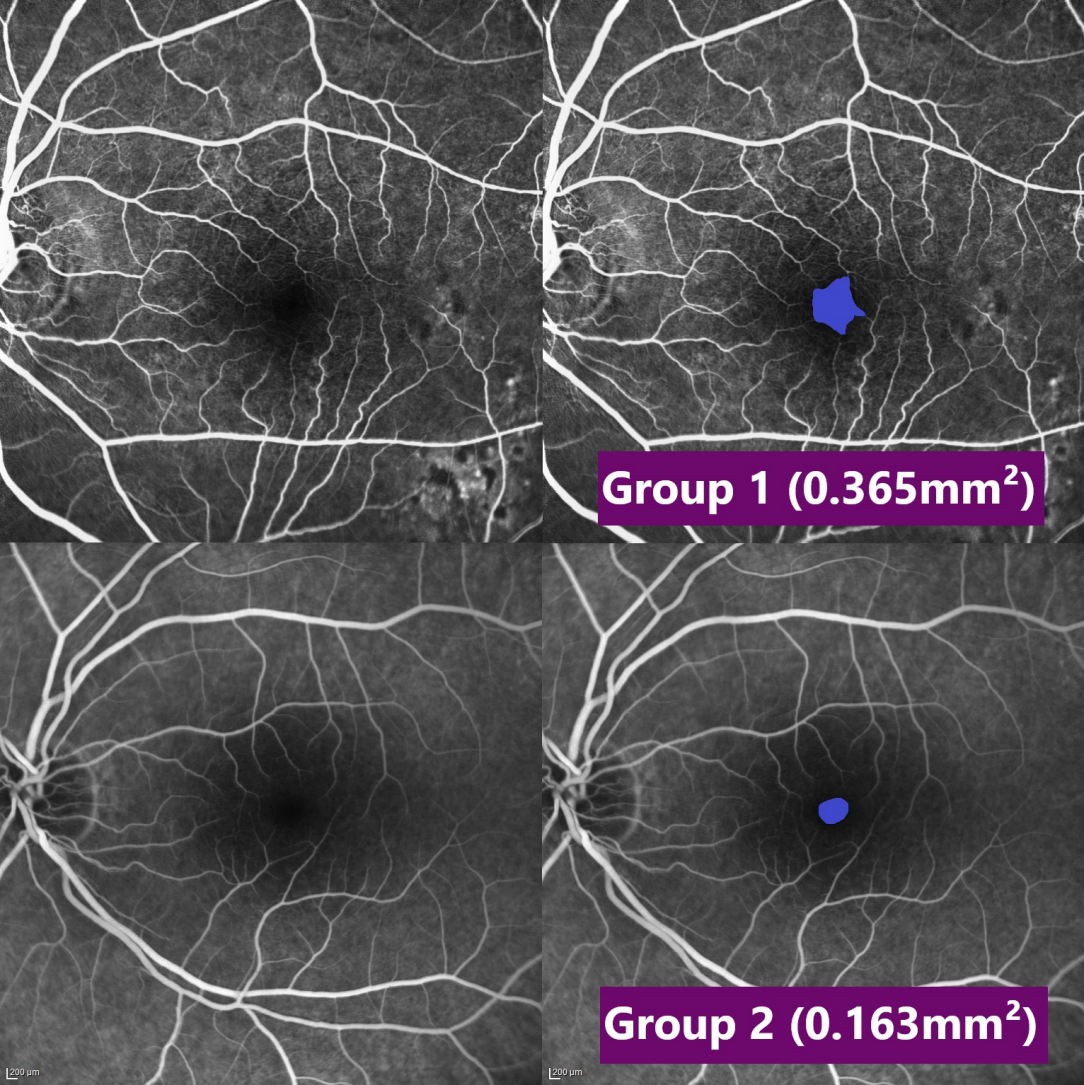
FAZ area. Exemplary early phase FA recordings of both groups from a matched pair without (left) and with (right) annotated FAZ area, showing a smaller FAZ area in the lower image (Group 2).

**Fig 5:**
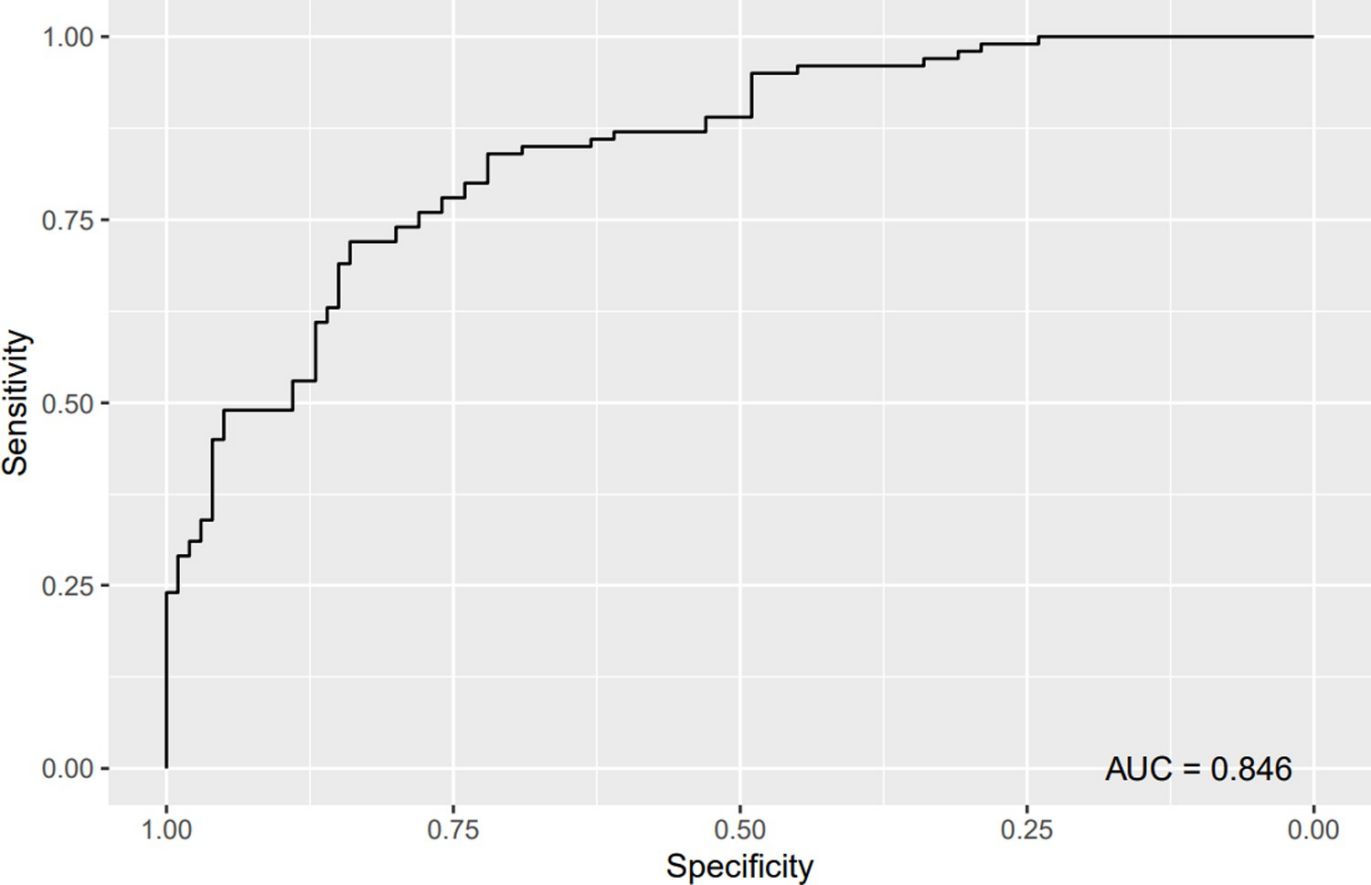
ROC-curve. ROC-curve of the conditional logistic model showing an excellent [19] prediction of cardiovascular related death (AUC = 0.846). The scale is described by Mandrekar [19] as follows: 0 indicates a perfectly inaccurate test, an AUC of 0.5 suggests no discrimination, 0.7 to 0.8 is considered acceptable, 0.8 to 0.9 is considered excellent, more than 0.9 is considered outstanding and a value of 1 reflects a perfectly accurate test.

**Fig 6:**
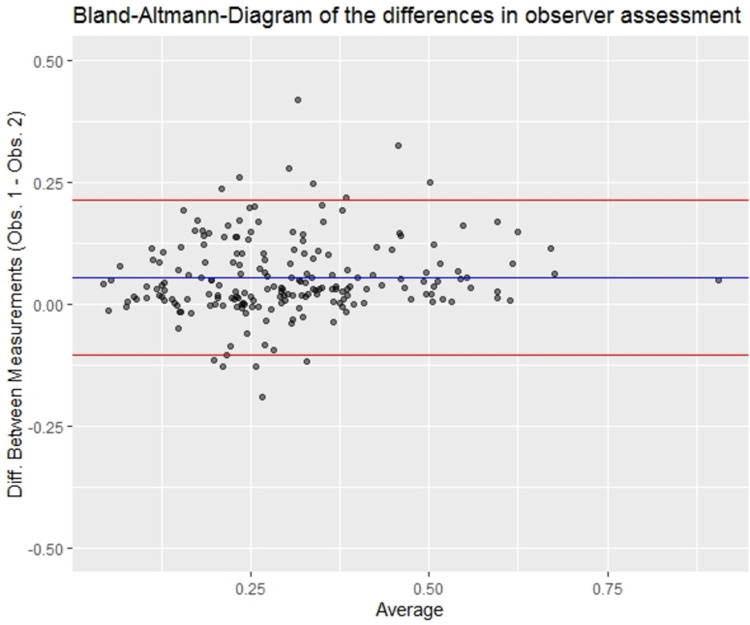
Bland-Altmann-Diagram. The Bland-Altmann-Diagram suggests a good agreement of the image graders that evaluated the size of the FAZ (ICC of 0.788).

Summary statistics for FAZ area, CRAE, CRVE and AVR are provided in Table 2. The mean FAZ area of our sample was 0.340 ± 0.135mm^2^ for Group 1 and 0.264 ± 0.137mm^2^ for Group 2 (p < 0.001), showing a larger FAZ area in patients who died of macrovascular-related events (Fig 4). There were no statistically significant differences in mean CRAE, mean CRVE and AVR between Group 1 and Group 2 (all p ≥ 0.523). In addition, summary statistics for FAZ area, CRAE, CRVE and AVR dependent on gender are provided in Table 3. There was a statistically significant difference in mean FAZ area in Group 1 and Group 2 considering both subgroups: female and male patients. Additionally, female patients of Group 1 showed a larger central retinal vein equivalent than female patients of Group 2 (p = 0.045). AVR of male patients was significant higher in Group 1 than in Group 2 (p = 0.042). Aside from that, no statistically significant differences were found in the subgroup analysis. In both the standard and the conditional logistic model, adjusted for confounders, FAZ area is considered significant (p = 0.002 and p = 0.005, respectively) which suggests it is correlated with later cardiovascular-related death (Table 4). In our data the models itself predict death from cardiovascular events moderately well (AUC_standard_ = 0.765 and AUC_conditional_ = 0.846 respectively). Note that the same data was used to compute the models and the ROC curves which means the goodness of the models is likely overrated (Fig 5). No systematic differences were found in the Bland-Altmann-Diagram (Fig 6). An ICC of 0.788 also suggests good observer consensus.

**Table 2:** Summary of fluorescein angiography measurements for Group 1 and Group 2.

	Group 1			Group 2				
	Mean	Min	Max	Mean	Min	Max	p Value	95% CI
Age (years)	69.66	39.44	82.15	69.39	38.84	81.99	-	-
FAZ area (mm^2^)	0.340	0.077	0.670	0.264	0.043	0.906	< 0.001 *	[-0.114; -0.038]
CRAE (μm)	187.98	127.53	240.28	185.89	108.02	250.01	0.523	[-4.37; 8.53]
CRVE (μm)	247.37	151.18	310.23	246.19	149.37	362.88	0.777	[-7.10; 9.47]
AVR	0.76	0.54	0.99	0.76	0.60	1.00	0.759	[-0.027; 0.02]

FAZ = Foveal avascular zone; CRAE = Central retinal arteriolar equivalent; CRVE = Central retinal venular equivalent; AVR = Arteriolar-Venular-Ratio; Statistically significant P values are indicated by an asterisk.

**Table 3:** Summary of fluorescein angiography measurements for Group 1 and Group 2 dependent on gender.

	Group 1		Group 2			
Female (n = 100)	Mean	SD	Mean	SD	p Value	95% CI
Age (years)	72.21	9.26	72.15	9.22	-	-
FAZ area (mm^2^)	0.333	0.14	0.272	0.14	0.042 *	[0.002; 0119]
CRAE (μm)	190.46	24.55	188.48	26.06	0.682	[-7.69; 11.66]
CRVE (μm)	249.27	27.30	238.63	28.63	0.045*	[-7.10; 9.47]
AVR	0.77	0.08	0.79	0.09	0.111	[-0.059; 0.006]
Male (n = 100)	Mean	SD	Mean	SD	p Value	95% CI
Age (years)	67.11	6.29	66.63	6.26	-	-
FAZ area (mm^2^)	0.347	0.13	0.256	0.15	0.001*	[0.042; 0.141]
CRAE (μm)	185.50	22.67	183.31	22.29	0.623	[-6.69; 11.06]
CRVE (μm)	245.48	28.00	253.75	34.48	0.198	[-21.00; 4.46]
AVR	0.76	0.10	0.73	0.08	0.042*	[0.001; 0.066]

FAZ = Foveal avascular zone; CRAE = Central retinal arteriolar equivalent; CRVE = Central retinal venular equivalent; AVR = Arteriolar-Venular-Ratio; Statistically significant P values are indicated by an asterisk.

**Table 4:** Results of the adjusted standard and conditional logistic regression model predicting death form cardiovascular-related disease.

	Standard logistic model				Conditional logistic model			
	Odds Ratio	Lower CL	Upper CL	p Value	Odds Ratio	LowerCL	UpperCL	p Value
FAZ Area	65.64	5.27	1003.09	**0.0017**	54.18	3.43	856.93	**0.0046**
DR	0.34	0.11	0.92	0.0429*	0.36	0.11	1.18	0.0905*
Diabetes	29.87	6.82	183.08	<0.001*	22.14	3.76	130.58	<0.001*
dAMD	2.85	0.70	14.63	0.1697*	1.95	0.34	11.08	0.4521*
nAMD	4.74	1.13	24.77	0.0448*	3.26	0.58	18.34	0.1808*
RVO	2.22	0.25	16.13	0.4348*	2.09	0.23	18.74	0.5083*
Glaucoma	2.18	0.43	12.24	0.3526*	2.17	0.34	13.93	0.4127*
Uveitis/Vasculitis	10.43	1.68	76.12	0.0144*	0.77	0.981	41.05	0.0877*
Other diagnose	10.16	2.49	53.28	0.0026*	6.62	1.25	35.09	0.0263*
No diagnose	5.19	0.78	37.68	0.0908*	4.41	0.54	36.16	0.1674*

FAZ = Foveal avascular zone; DR = Diabetic Retinopathy as graded by Retinal Specialist on fluorescein angiography, dAMD = Dry age-related macular degeneration, nAMD = neovascular age-related macular degeneration, RVO = Retinal vein occlusion.

* p values only for descriptive use, multiple testing corrections were not performed.

## Discussion

In this retrospective matched case-control study, we examined the FAZ area and vessel calibers in patients who died of cardiovascular diseases and individuals who have still been alive in 12/2018 using fluorescein angiography. We could show that an enlarged FAZ area can predict CVD death in men and women aged 39–82 years moderately well. After subgroup analysis, we found that increased retinal venular caliber was associated with CVD related mortality in women, but not in men.

In the last decades the FAZ was an essential part in numerous clinical-ophthalmic scientific studies and its functional correlation with various ocular diseases was examined [20]. Prior studies have shown that an increased area of the FAZ is correlated with impaired visual acuity (VA) in patients with diabetic retinopathy (DR) and retinal vein occlusion (RVO) [21–23]. A reduction in perfusion due diabetic angiopathy or vascular occlusion may lead to ischemia in the fovea which has an important effect on VA [24]. In addition Li et al. have shown that an enlarged FAZ area is detectable in diabetes patients before clinical signs of DR occur [10]. This suggest that the FAZ is highly sensitive to ischemic events and could be a good predictor for alterations in the retinal microvasculature even before consequential damages are visible.

In this study we could demonstrate that the FAZ area is significantly increased in patients whose death was caused by a CVD during lifetime FA examinations in comparison to matched controls. Even after a subgroup analysis the same results could be shown for men and women independently. Similar to our control group John et al. [25] determined in a study with 31 healthy individuals a mean FAZ area of 0.275±0.074 mm^2^. Enlargement of the FAZ was already described in previous studies for different diseases, including DR [26], macular branch retinal vein occlusion [27], sickle cell disease [28], talc retinopathy [29] and arterial hypertension [12, 13]. Especially DR has a strong association with CVD. It stands to reason considering that diabetes patients are two to four times more likely to develop CVD than people without diabetes. DR and CVD patients share several risk factors, such as age, duration of diabetes, hypertension, higher BMI and chronic kidney disease [30]. We assume that a certain proportion of our study population suffered from diabetes and/or exhibited some of the risk factors mentioned. Due to the nature of a retrospective study, we cannot determine which disease or risk factor had the greatest impact on the FAZ enlargement. However, we could show that the FAZ can predict death from cardiovascular events acceptably [19] well (AUC = 0.846) and it may not be important if diabetes is the primary cause for the FAZ enlargement or if the reason for FAZ enlargement is elsewhere and as such already a predictor for a potential cardiovascular event. Thus, it would be interesting to investigate the same study in just diabetic patients in the future only. These results spark hopes that the FAZ could become an important imaging biomarker for the risk assessment of cardiovascular mortality.

Regarding the other retinal imaging biomarker, including arteriolar and venular caliber and the arteriolar-venular-ratio, we could not demonstrate statistically significant differences between patients who died of CVDs and patients who did not. However, previous studies have documented that retinal vessel caliber changes can be a predictive marker for CVDs [31, 32]. Wang et al. have shown that a larger retinal venular caliber is associated with a 1.5-2x increased risk of coronary heart disease (CHD) death in men and women aged 49–75, but a smaller arteriolar caliber only predicted higher risk of fatal CHD in women [33]. Furthermore McGeecha et al. demonstrated that a wider retinal venular caliber is independently associated with an increased risk of stroke events [34]. One possible explanation for our results could be that FA images are not as well suited as color fundus images to measure vessel diameters. Due to the fluorescent dye, vessel calibers could appear larger than they are. This influence could be different strong for arterioles and venules due to the varying contrast agent filling during the FA examination. Nevertheless, after subgroup analysis, we could show that increased retinal venular caliber was associated with CVD related deaths in women, but not in men. This finding substantiates the hypothesis that microvascular coronary artery disease may be more prominent among women [35, 36].

Despite the increasing predominance of evidence of a connection between retinal microvascular alterations and CVDs, there still remains a substantial lack of understanding about the pathophysiologic mechanisms underlying the relationship between microvascular and macrovascular disease [8]. The central pathological mechanism in CVD is atherosclerosis and is thought to result from endothelial injury and chronic inflammation [37]. The net effect of this process is the formation of a fibro-lipid plaque with a fibrous cap in the peripheral or coronary vascular system. Rupture of this atheroma results in an acute vascular infarction which could lead to myocardial infraction or stroke. Contrary to this, microvascular complications for example in diabetes can result in retinopathy, nephropathy, or neuropathy. Several studies have tried to substantiate the relationship between retinal microvascular abnormalities and atherosclerosis. It was suggested that extent and severity of retinal vessel atherosclerosis is strongly correlated with the extent and severity of coronary artery disease (CAD) [38, 39]. Van Hecke et al. hypothesized that microvascular changes may be linked to atherosclerosis by inducing endothelial dysfunction of large vessels. But their study showed that retinal microvascular disease was not independently associated with endothelial-dependent flow-mediated vasodilation (reflects impaired endothelial function) or early atherosclerosis [40]. In contrast, Wu et al. examined in a relatively small study the association of central retinal artery (CRA) blood flow and endothelial-dependent flow-mediated vasodilation. They demonstrated that increased CRA resistance and decreased CRA blood flow was correlated with decreased brachial artery endothelial function in CAD patients, indicating a connection between retinal vascular changes and endothelial dysfunction [41]. However, endothelial dysfunction for example in diabetes can be caused by several metabolic insults that include hyperglycemia, dyslipidemia, hypertension, hyperinsulinemia, elevated levels of fatty acids, increased production of reactive oxygen species, hyperleptinemia, cytokine‐mediated inflammation, activation of the sympathoadrenal system, activation of protein kinase C and the induction of a prothrombotic diathesis [42].

In summary, the connection between microvascular disease and CVD appears to be multifactorial, bidirectional, and dependent on mutual risk factors such as diabetes and arterial hypertension. It remains uncertain which micro- or macrovascular alteration is cause and which is effect, but endothelial dysfunction seems to play a critical role. Nevertheless, the underlying pathophysiology between micro- and macrovascular disease is not fully resolved and further research is required.

Strengths of our study include precise assessment of retinal microvascular biomarkers by experienced image graders of the Vienna Reading Center and tracing of CVD deaths by validated Austrian death registry data. On the other hand, limitations of our study were: Patients who did not die of cardiovascular vascular disease cannot be considered “healthy”. Some of them suffer from internal diseases, including even cardiovascular diseases. Those diseases certainly also affected the retinal microvasculature of our control cohort. Since medical records were not always available, we could only adjust our results for CVD risk factors such as diabetes and the assigned ocular diagnoses. Despite that we matched for gender and age, a selection bias of unmatched case-controls regarding gender-balanced CVD risk factors could be possible. In addition, 140 patients who had died of CVD and 98 patients who are still alive did not have gradable FA examinations for the assessment of microvascular biomarkers. This is inevitable when real world data is used and might have led to a selection bias. However, exclusion criteria were applied equally and strictly to both groups. Finally, the number of CVD deaths was relatively small, and our findings need confirmation in larger studies. This study is a proof of principle hypothesis building retrospective analysis, which aimed at identifying potential imaging biomarkers for the risk assessment of cardiovascular mortality. In later prospective clinical studies, it will be necessary to validate the impact of FAZ measurement in addition to traditional CVD risk factors using non-invasive imaging methods and controlling for potential confounders.

With the increasing use of OCTA devices in clinical routine, there is a noninvasive, non-harmful and fast opportunity to capture the retinal microvasculature. In contrast to FA, OCTA provides a detailed angiographic representation of the microvasculature, allowing qualitative and quantitative analysis of the perfusion state of individual retinal capillary plexuses of the fovea, unobscured by leakage. In many departments automated and semi-automated measurements of the FAZ using OCTA images are already established. This implies that early detection of retinal microvascular alterations (e.g., seen as enlargement of the FAZ size) is viable and could extend traditional risk factors of CVDs. This might help to initiate prevention strategies just in time, preventing and reducing the burden of cardiovascular mortality. Certainly, it will be needed to prove our suggestions in a prospective clinical trial before conclusion are valid. Monteiro-Henriques et al. recently concluded that OCTA could be a useful tool to assess a patient’s CVD risk profile in a non‐invasive way [43], therefore using OCTA in such a trial will be the best strategy and will also lead to less exclusions of patients as seen in FA, e.g. due to leakage or blocked fluorescein.

In conclusion, in this retrospective matched case-control study, we demonstrated with clinical routine FA data that patients whose death was caused by a macrovascular-related disease show on average a larger FAZ area on FA examinations during lifetime than patients who are alive. Our findings substantiate the hypothesis of an association between retinal microvascular alterations and CVD.

## Supporting information

S1 Data (XLSX)
